# A nomogram model based on preoperative grey-scale US features and routine serum biomarkers to predict the outcome of infants with biliary atresia after Kasai portoenterostomy

**DOI:** 10.3389/fped.2022.972855

**Published:** 2022-10-20

**Authors:** Zongjie Weng, Fengying Ye, Luyao Zhou, Fa Chen, Wen Ling, Yifan Fang, Min Liu, Qiumei Wu, Xiuqing Qiu, Guorong Lyu

**Affiliations:** ^1^ Department of Medical Ultrasonics, The Second Affiliated Hospital of Fujian Medical University, Quanzhou, China; ^2^ Department of Medical Ultrasonics, Fujian Maternity and Child Health Hospital, College of Clinical Medicine for Obstetrics / Gynecology and Pediatrics, Fujian Medical University, Fuzhou, China; ^3^ Department of Medical Ultrasonics, Institute for Diagnostic and Interventional Ultrasound, the First Affiliated Hospital, Sun Yat-Sen University, Guangzhou, China; ^4^ Department of Epidemiology and Health Statistics, School of Public Health, Fujian Medical University, Fuzhou, China; ^5^ Department of Pediatric Surgery, Fujian Maternity and Child Health Hospital, College of Clinical Medicine for Obstetrics / Gynecology and Pediatrics, Fujian Medical University, Fuzhou, China; ^6^ Department of Gynecology, Fujian Maternity and Child Health Hospital, College of Clinical Medicine for Obstetrics / Gynecology and Pediatrics, Fujian Medical University, Fuzhou, China; ^7^ Department of Clinical Medicine, Quanzhou Medical College, Quanzhou, China

**Keywords:** biliary taresia, Kasai portoenterostomy, nomogram, hilar lymph nodes, prognostic prediction

## Abstract

**Objective:**

To establish a nomogram to predict the outcome of biliary atresia (BA) infants 3-months post- Kasai portoenterostomy (KPE).

**Methods:**

BA Infants who underwent KPE from two hospitals were included in the training (*n* = 161) and validation cohorts (*n* = 64). A logistic regression equation (Equation A) for predicting the serum total bilirubin (TBIL) level 3-month post-KPE was established in the training cohort. Then, a nomogram was developed based on Equation A in the training cohort and validated in the validation cohort. Moreover, a new equation (Equation B) was generated based on the nomogram and the size of the enlarged hilar lymph nodes (LNs) in the validation cohort. The predictive performance of the nomogram was evaluated by the receiver operating characteristic (ROC) curve and by calculating the area under the ROC curve (AUC), sensitivity, specificity, and positive (PPV) and negative (NPV) prediction values.

**Results:**

A nomogram based on gallbladder morphology and serum levels of TBIL and total protein (TP) was established with AUC (95%CI) of 0.673 (0.595, 0.745) and 0.647 (0.518, 0.763), sensitivity (95%CI) of 71.4% (62.1%,79.6%) and 81.8% (59.7%,94.8%), specificity (95%CI) of 63.3% (48.3%,76.6%) and 47.6% (32.0%,63.6%), PPV (95%CI) of 81.6% (72.5%,88.9%) and 45.0% (29.3%,61.5%), and NPV (95%CI) 49.2% (36.4%,62.1%) and 83.3% (62.6%,95.3%), respectively, in the training and validation cohorts. Furthermore, in the validation cohort, the AUC (95%CI) of Equation B was 0.798 (95%CI: 0.679, 0.888), which was significantly higher than that of the nomogram (*P *= 0.042).

**Conclusion:**

A nomogram based on the pre-KPE gallbladder morphology, TBIL, and TP to predict the outcome of BA 3-months post-KPE is established. Moreover, the addition of the size of the enlarged hilar LNs into the nomogram further improves its predictive value.

## Introduction

Biliary atresia (BA) is a rare condition that occurs in approximately 1/5,000–1/19,000 live births (1–3) and remains the most common indication for liver transplantation in children (4). If infants with BA are left untreated, progressive liver cirrhosis will lead to death by the age of 2 years (5). Kasai portoenterostomy (KPE), proposed by Kasai in 1959 (6), has evolved into the standard procedure for BA. Traditional KPE is open surgery. A few years ago, laparoscopic Kasai procedure was reported to be feasible (7–10). However, many studies have proven the efficacy of laparoscopic Kasai to be unfavorable (11–14). Therefore, open KPE remains the first-line surgical modality to restore bile flow in infants with BA (15, 16).

Clinically, the outcome of KPE is evaluated by the time of native liver survival (NLS). According to the statistics (17–20), the 5-year, 10-year, and overall NLS rates range from 32% to 59%, 27% to 52%, and 42% to 89%, respectively. Shneider et al*.* (21) reported that there is a clear difference in NLS between children with serum total bilirubin (TBIL) <2 mg/dl and those with TBIL >6 mg/dl at 3 months after KPE. Recent studies (22–25) have demonstrated that serum TBIL <2 mg/dl at 3 months after KPE is a surrogate predictor for favorable NLS or satisfactory KPE efficacy.

Even KPE can relieve symptom of biliary obstruction, it can not stop the process of immune-mediated inflammatory processes which may be one of the causative factors of BA. Therefore, it's still possible to appear many complications after surgery, including postoperative fever, jaundice, recurrent cholangitis, and rapidly progressive liver cirrhosis, which may consequently lead to unsatisfied prognosis, including liver failure, death or liver transplantation. If there is a method to predict unsatisfactory surgical outcomes before KPE, it can help doctors choose better clinical treatment or prevention methods.

Many studies have tried to identify the outcome predictors after KPE. Several studies (26–30) reported that pre-KPE liver biopsy and elastography can predict KPE outcome by evaluating liver degree. However, although liver biopsy is the gold standard for fibrosis, it is invasive and unsafe for BA infants, and the obtained histological material is insufficient for an appropriate analysis (27). Elastography is noninvasive, but it is not equipped in most of ultrasonic devices, which limits its clinical application. In addition, a preliminary study (5) demonstrated that high levels of serum basic fibroblast growth factor and hepatocyte growth factor prior to KPE surgery were associated with a poor KPE prognosis, whereas decreased concentrations of serum transforming growth factor-β and epidermal growth factor were associated with a good outcome. However, these special tests are not only expensive and time-consuming, but also hard to apply in clinical practice, especially in primary hospitals. Recently, a few studies reported that age at operation (31, 32), the level of direct bilirubin (DBIL) (33–36), aspartate aminotransferase (AST) (35, 36), and γ-glutamyltransferase (GGT) (37) could predict the outcome of KPE. However, these parameters were usually analyzed individually. Therefore, a more accurate method that combines these routine laboratory parameters with another non-invasive technique is required for prognosis prediction of infants with BA.

Grey-scale ultrasonography (US) is the most popular imaging tool for the diagnosis of BA (38, 39). The gallbladder morphology, triangular cord (TC) thickness, and hilar lymph nodes (LNs) are three major features used for BA diagnosis on US, with gallbladder morphology and TC thickness being the most commonly used US indicators (38–48) as they directly reflect biliary system abnormalities. We proposed that pre-KPE US assessment of these two features is valuable for KPE outcome prediction.

Therefore, the objective of the present study was to establish a nomogram model based on preoperative grey-scale US features and routine serum biomarkers to predict the outcome of BA infants 3-months post-KPE.

## Materials and methods

### Patients

Infants who suffered from BA and underwent KPE at the First Affiliated Hospital of Sun Yat-Sen University between January 2003 and December 2016 (allocated into the training cohort) and the Fujian Provincial Maternity and Children's Hospital between January 2012 and December 2021 (allocated into the validation cohort) were included in this retrospective study. Infants were excluded according to the following criteria: (1) there was no transabdominal US examination within one week before KPE or the US images were uncompleted or unclear; (2) there were no routine serum biomarker examinations (blood routine biomarkers, liver function biomarkers) within one week before KPE; and (3) there were no routine serum biomarker examinations 3 months after KPE.

The Institutional Review Board and Ethics Committee of the First Affiliated Hospital of Sun Yat-Sen University and the Fujian Provincial Maternity and Children's Hospital approved the study protocol and waived the need for parents' informed consent due to the retrospective nature of the study design.

### US examination

The US devices used were a Voluson E8 scanner (GE Medical System, Boston, the U.S.A) equipped with a 4–8 MHz curvilinear transducer and a 11 MHz linear array transducer, an SSA-660A scanner (Toshiba Medical Systems, Tokyo, Japan) equipped with a 3.5 MHz curvilinear transducer and a 12 MHz linear array transducer, an EUB-7000HV scanner (Hitachi Medical Corporation, Tokyo, Japan) equipped with a 2–5-MHz curvilinear transducer and a 6–13 MHz linear array transducer, and an Aixplorer scanner (Supersonic, Paris, France) equipped with a 1–6 MHz curvilinear transducer and a 4–15 MHz linear array transducer.

Infants were not fed for at least 4 h prior to US examination. During the examination, they were kept quiet and if required, fed with milk during the examination. The high-frequency transducers (>10 MHz) were used for the examination. First, the gallbladder fossa was detected carefully to determine the presence of the gallbladder. If the gallbladder was detected, its length, lumen, outline, wall, and mucosal lining were evaluated, and at least one long axis section image was saved. Then, the TC thickness was measured, and at least one image was saved.

At Fujian Provincial Maternity and Children’s Hospital, the hepatic hilar was also scanned to determine the presence of LNs. If LNs were detected, at least one long axis section image was saved, and the maximal length of the largest hepatic hilar LN was measured.

### US images analysis

The US images were retrospectively reviewed by two radiologists (L.Y.Z. and Z.J.W with 12 and 9 years of experience in pediatric US, respectively). The final conclusions of images were given by the two radiologists after consultation. The two radiologists were blind to the other clinical data before completing image analysis.

**Figure 1:**
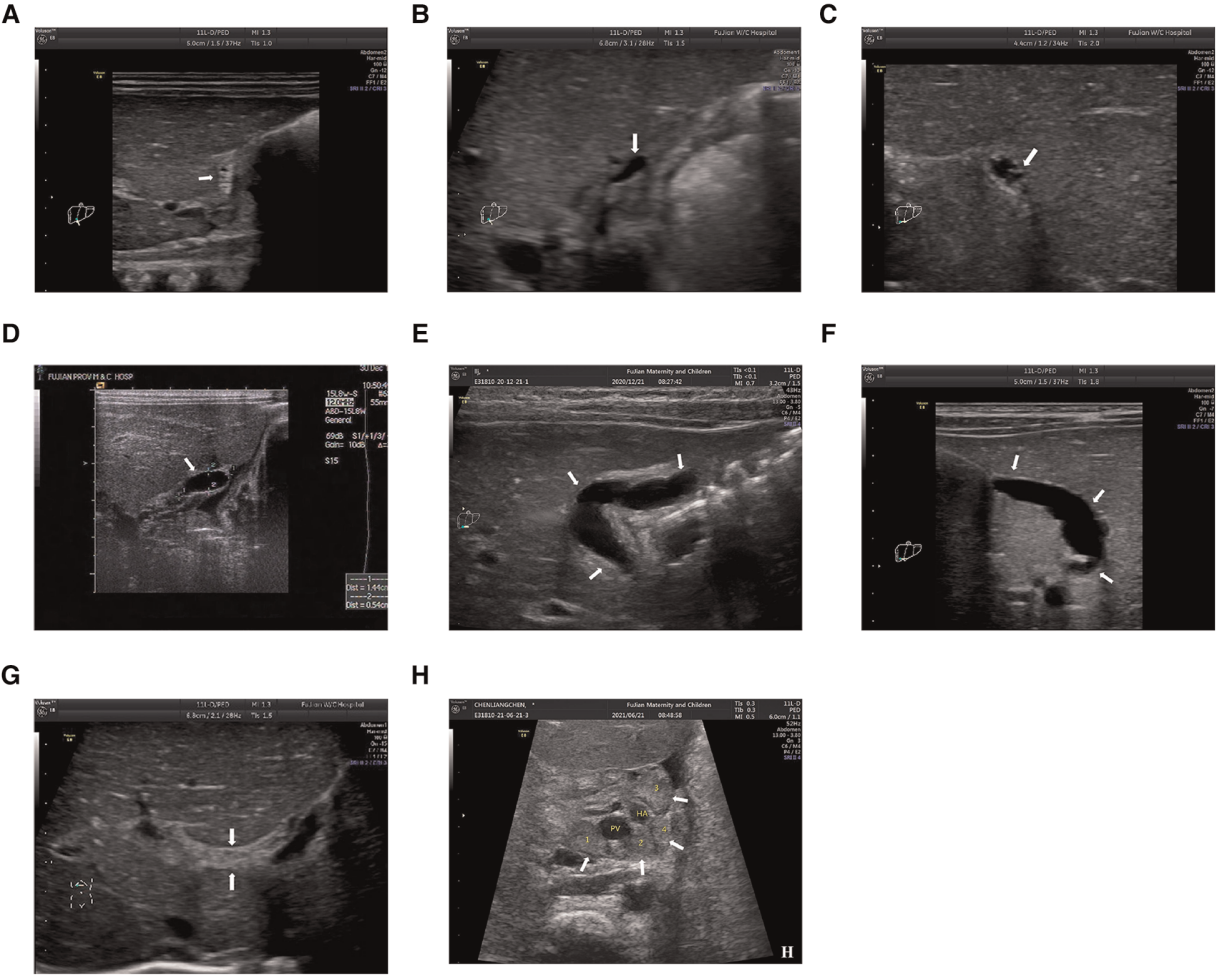
(**A**) the gallbladder is not identifiable; (**B**) the length is less than 1.5 cm; (**C**) the gallbladder is with irregular outline; (**D**) the wall of gallbladder is not identifiable, and the gallbladder appears as a cyst in the gallbladder fossa; (**E**) the gallbladder has an irregular wall thickness; (**F**) the hyperechogenic mucosal lining of gallbladder is not smooth; (**G**) the triangular cord (TC) thickness is the thickness of the echogenic anterior wall of the anterior branch of the right portal vein; and (**H**) the hilar lymph nodes (LNs) are detected around porta hepatis. PV, portal vein; HA, hepatic artery. The arrows in (**A–F**) indicate the location of the gallbladder; The arrows in (**G**) indicate the location of TC thickness; The arrows in (**H**) indicate the location of hilar LNs.

The gallbladder morphology was classified into abnormal and normal according to previous studies (40, 41, 43, 48, 49). The gallbladder was considered abnormal if at least one of the following criteria was met: (1) The gallbladder morphology was not identified; (2) The length of gallbladder was less than 1.5 cm; (3) The outline of gallbladder was irregular; (4) The wall of gallbladder was not identifiable, and the gallbladder appeared as a cyst in the gallbladder fossa; (5) The wall thickness of gallbladder was irregular; and (6) The hyperechogenic mucosal lining of gallbladder was not smooth or incomplete (Figures 1A–F). The gallbladder morphology was considered normal if one of the following criteria was met: (1) the gallbladder was detected without a lumen, but a smooth and complete hyperechogenic mucosal lining was visualized and the wall was uniformly thickened or the lumen was incompletely filled with a smooth and complete hyperechogenic mucosal lining with the walls in part in close approximation; or (2) the gallbladder was detected with a fully filled lumen and lumen length more than 1.5 cm without wall thickening, and the hyperechogenic mucosal lining of the gallbladder was smooth.

The TC thickness was defined as the thickness of the echogenic anterior wall of the anterior branch of the right portal vein (PV) distal to the right PV on a longitudinal image, without including the right hepatic artery, according to Lee et al. (50) (Figure 1G). The cut-off value of the TC thickness diameter was set to 2 mm according to our previous study (43).

Previous studies (51, 52) have demonstrated that the size of hilar LNs could reflect the severity of cholestasis and thus might reflect the severity of fibro-obliterative bile duct lesion to some extent. Therefore, we hypothesize that hilar LNs on US scan could also be used for prognostic prediction. In the present study, the hilar LNs were only detected on patients admitted to the Fujian Provincial Maternity and Children's Hospital (validation cohort) because hilar LNs were a newly proposed US feature first mentioned in 2019 and thus the data on hilar LNs for the diagnosis of BA were absent in the training cohort. Specifically, hilar LNs were detected around the porta hepatis in front of the PV. The maximal diameter of each LN was measured at least twice for one LN, and the mean value was used for further analysis. Finally, the largest mean value was adopted when there was more than one LN (Figure 1H).

### Routine serum biomarkers

Routine laboratory serum biomarkers detected within one week before KPE, including the percentage of neutrophils (NE%), absolute value of neutrophils (NE#), hemoglobin (HGB), platelet (PLT), TBIL, DBIL, indirect bilirubin (IBIL), total bile acid (TBA), total protein (TP), albumin (ALB), globulin (GLB), alanine transaminase (ALT), AST, and GGT, were included in the analysis.

### KPE and follow-up after KPE

Open KPE was performed as previously described (6). After discharge, patients were followed up once a month and monitored by the outpatient visit for at least three months. The outcome of patients was defined as satisfactory (TBIL ≤ 2 mg/dl) or unsatisfactory (TBIL > 2 mg/dl) according to the serum TBIL level 3-month after KPE (21–25).

### Statistical analysis

Continuous variables were expressed as the mean ± standard deviation (SD) or the median with interquartile range (IQR) (25th–75th percentile) and were compared by using the *t*-test or Mann–Whitney *U* test, where appropriate. Categorical variables were expressed as the percentage and compared using the *χ*^2^ test or Fisher's exact test, where appropriate. In addition, continuous variables except for the TC thickness were transformed into categorical variables according to the cut-off value determined by the receiver-operating characteristic (ROC) curves.

In the training cohort, univariate logistic regression was used to evaluate the association of the potential predictive factors with the outcome of KPE. All variables with *P*-value less than 0.2 were chosen as candidates for the subsequent multivariate logistic regression analysis. A logistic regression equation (Equation A) was generated, and a nomogram was then developed using the “rms” package of R software. Each variable in the nomogram was given a corresponding weighted score based on its β coefficient in Equation A. The predictive performance of the nomogram was evaluated by the concordance index (C-index) and calibration curve with 1,000 times bootstrap resampling. The ROC curve of nomogram was plotted, and the area under curve (AUC), sensitivity, and specificity were calculated. In addition, a 95% confidence interval (CI) was also calculated where required.

In the validation cohort, the AUC, sensitivity, and specificity of the nomogram developed in the training cohort were calculated in the validation cohort. Moreover, a new equation (Equation B) was generated based on the nomogram and a newly added variable, the size of enlarged hilar LNs, by using multivariate logistic regression analysis. Then, the ROC curve of Equation B was further drawn in the validation cohort, and the AUC of Equation B were compared with that of the nomogram calculated in the validation cohort by DeLong's test.

Statistical analyses were performed with statistical software SPSS, version 16 (IBM Corp, New York, USA) and R, version 4.1.1 (R Development Core Team). Statistically significant difference was defined as a *P*-value <0.05.

## Results

### Baseline clinical characteristics

The training cohort enrolled 161 infants from the First Affiliated Hospital of Sun Yat-Sen University, and the validation cohort included 64 infants from Fujian Provincial Maternity and Children's Hospital. Of these, 49 (30.4%) and 22 (34.4%) infants achieved satisfactory KPE outcome (TBIL ≤ 2 mg/dl) in the training cohort and validation cohort, respectively.

The baseline clinical characteristics of the patients are listed in Table 1. There was no difference between the two cohorts in the variables except for the prevalence of patients with gallbladder abnormalities, NE%, PLT, DBIL and TBA (all *P *< 0.01).

**Table 1:** Baseline clinical characteristics of infants with biliary atresia.

Variable	Cohort		*P-*value
	Training cohort (*n* = 161)	Validation cohort (*n* = 64)	
Age at operation, day	65 (57,78)	67 (55,81)	0.810
Sex, No (%)			0.916
Male	80 (49.7%)	32 (50%)	
Female	81 (50.3%)	32 (50%)	
Gallbladder morphology *n*, No (%)			<0.01
Normal	41 (25.5%)	6 (9.4%)	
Abnormal	120 (74.5%)	58 (90.6)	
TC thickness, mm	2.4 (1.6,3.0)	2.8 (0,3.5)	0.378
NE%, %	23.4 (19.1,30.1)	29.0 (23.3,36.6)	<0.01
NE#, 10^9^/L	2.8 (2.1,3.6)	2.9 (2.3,4.9)	0.108
HGB, g/L	103.0 (97.0,111.0)	105.5 (96.0,120.8)	0.309
PLT, 10^9^/L	450.5 (379.5,551.0)	386.0 (310.8,496.5)	<0.01
TBIL, µmol/L	161.6 (133.3,192.8)	166.8 (139.3,206.0)	0.208
DBIL, µmol/L	112.2 (98.5,130.4)	122.2 (98.2,146.7)	0.051
IBIL, µmol/L	41.9 (30.1,63.1)	38.8 (30.3,55.6)	0.606
TBA, µmol/L	136.7 (112.5,162.2)	86.9 (67.4,105.2)	< 0.01
TP, g/L	57.0 (53.2,59.8)	52.0 (49.2,55.6)	0.786
ALB, g/L	39.0 (37.0,41.2)	36.8 (34.9,39.0)	0.064
GLB, g/L	17.0 (14.9,19.9)	16.6 (13.9,18.5)	0.081
ALT, U/L	123.0 (85.0,178.0)	147.3 (91.8,234.5)	0.061
AST, U/L	199.0 (138.8,280.3)	201.3 (149.5,280.2)	0.962
GGT, U/L	606.5 (339.5,962.0)	600.0 (284.3,843.8)	0.165

Data are expressed as the median (interquartile range) or number (%), where appropriate.

TC thickness, triangular cord thickness; NE%, percentage of neutrophils; NE#, absolute value of neutrophils; HGB, hemoglobin; PLT, platelet; TBIL, total bilirubin; DBIL, direct bilirubin; TBA, Total bile acid; TP, total protein; ALB, albumin; GLB, globulin; ALT, alanine transaminase; AST, aspartate aminotransferase; GGT, *γ*-glutamyltransferase.

### Establishment of the prognostic prediction nomogram in the training cohort

In the training cohort, seven variables with *P*-values less than 0.2 in univariate logistic regression analysis were selected for the subsequent multivariate logistic regression analysis (Table 2). The result of multivariate logistic regression analysis was shown in Table 2. The gallbladder morphology, TBIL, and TP were significantly associated with the unsatisfactory outcome of KPE.

**Table 2:** Univariate and multivariate logistic regression analyses in the training cohort.

	Univariate logistic regression					Multivariate logistic regression				
	B	S.E	Wald	*P*	OR	B	S.E	Wald	*P*-value	OR
Gallbladder morphology	0.813	0.454	3.215	0.073	2.256	1.157	0.534	4.701	0.030	3.180
TBIL	0.846	0.355	5.667	0.017	2.330	1.000	0.498	4.034	0.045	2.719
TP	0.549	0.372	2.172	0.141	1.731	1.267	0.474	7.147	0.008	3.520
ALT	1.093	0.781	1.962	0.161	2.985					
AST	−0.673	0.532	1.603	0.198	0.510					
GGT	0.715	0.366	3.811	0.051	2.043					
DBIL	0.114	0.369	2.098	0.158	1.121					
Constant						−1.161	1.000	1.349	0.245	0.313

B, β coefficient; SE, standard error; Wald, *χ*^2^ value of Wald test; OR, odds ratio; TBIL, total bilirubin; TP, total protein; ALT, alanine transaminase; AST, aspartate aminotransferase; GGT, γ-glutamyltransferase; DBIL, direct bilirubin.

The final regression equation (Equation A) that was derived from these three risk factors was formatted as follows: Log(*p*) = −1.161 + 1.157 × (Gallbladder morphology) + 1.000 × (TBIL) + 1.267 × (TP). The *P* is the probability of achieving unsatisfactory outcome (i.e., TBIL> 2 mg/dl). In Equation A, the gallbladder morphology was defined as 0 when it was normal and as 1 when it was abnormal; the TBIL level was defined as 0 when it was ≤157.3 µmol/l and as 1 when it was >157.3 µmol/l; and the TP level was defined as 0 when it was >55 g/L and as 1 when it was ≤55 g/L.

**Figure 2:**
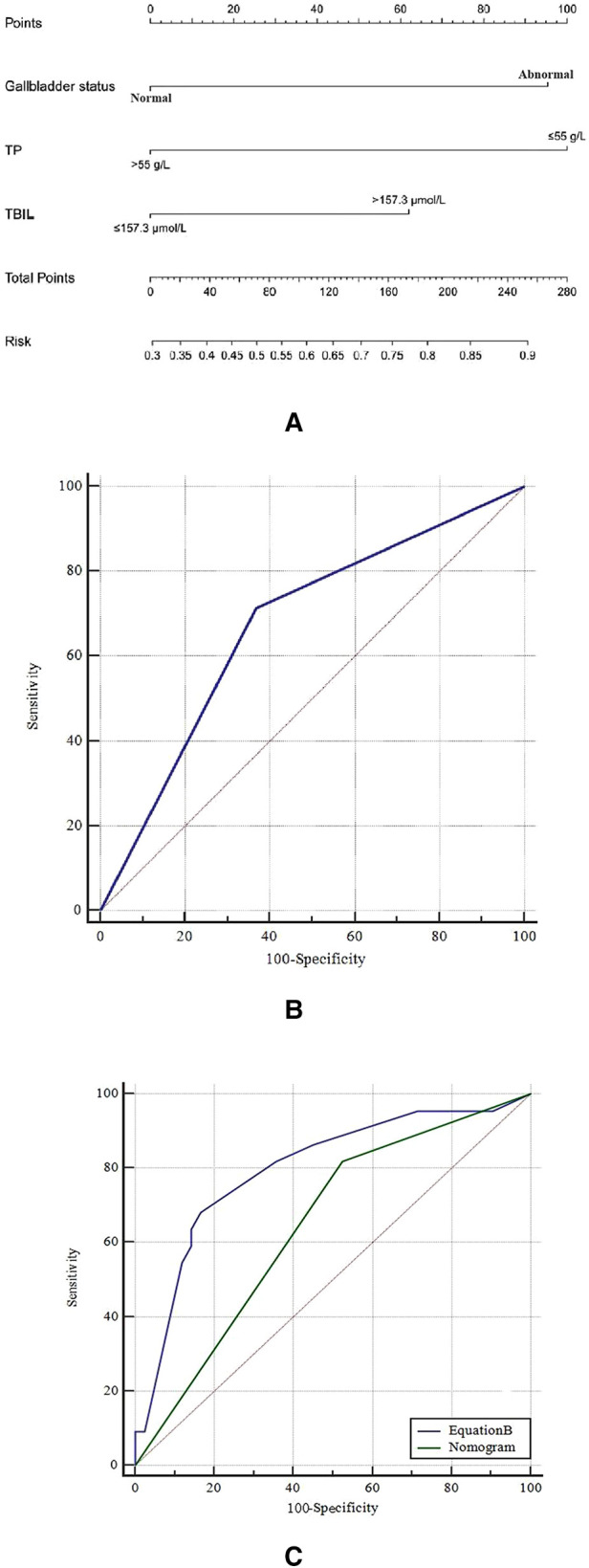
(**A**) the nomogram in the training cohort; (**B**) the receiver operating characteristic (ROC) curve of the nomogram in the training cohort; and (**C**) the comparison of ROC curve between the nomogram and equation B in the validation cohort. TP, total protein; TBIL, total bilirubin.

Then, a nomogram model was developed based on Equation A (Figure 2A); each of the three predictors was given a corresponding weighted score based on its β coefficient in Equation A, and the estimated probability of achieving unsatisfactory outcome (i.e., TBIL > 2 mg/dl) was determined by the sum of the scores of the three predictors. Specifically, the gallbladder morphology was scored 0 points when it was normal and 95 points when it was abnormal; the TBIL level was scored as 0 points when it was ≤157.3 µmol/l and 62 points when it was >157.3 µmol/l; and the TP level was scored 0 points when it was >55 g/L and 100 points when it was ≤55 g/L.

The nomogram demonstrated good ability in predicting the outcome of KPE, with an unadjusted C-index of 0.680 (95%CI: 0.664, 0.696) in the training cohort and 0.730 (95%CI: 0.699, 0.761) in the validation cohort. The calibration curves in the training and validation cohorts revealed that the predicted probabilities by the nomogram were a good match with the actual observations. The ROC curve of the nomogram model in the training cohort is shown in Figure 2B, with the Youden's index being the largest when the sum of the total points was 100. Therefore, the sum of the total points of 100 was taken as a cut-off value; a sum of the total points of >100 predicted an unsatisfactory outcome (i.e., TBIL > 2 mg/dl). The ROC curve of the nomogram model in the validation cohort is illustrated in Figure 2C.

### The predictive performance of the nomogram in the training and validation cohorts

In the training cohort, the AUC was 0.673 (95% CI: 0.595, 0.745), and the sensitivity, specificity, positive prediction value (PPV), and negative prediction value (NPV) of the nomogram model were 71.4% (95% CI: 62.1%, 79.6%), 63.3% (95% CI: 48.3%, 76.6%), 81.6% (95% CI: 72.5, 88.7), and 49.2% (95% CI: 36.4%, 62.1%), respectively (Table 3).

**Table 3:** The prediction performance of nomogram model in training and validation cohorts.

	Value (95%CI)	
	Training cohort	Validation cohort
Cut-off value	>100	>100
AUC	0.673 (0.595, 0.745)	0.647 (0.518, 0.763)
Sensitivity (%)	71.4 (62.1,79.6)	81.8 (59.7,94.8)
Specificity (%)	63.3 (48.3,76.6)	47.6 (32.0,63.6)
Positive predictive value (%)	81.6 (72.5,88.9)	45.0 (29.3,61.5)
Negative predictive value (%)	49.2 (36.4,62.1)	83.3 (62.6,95.3)

CI, confidence interval; AUC, are under the curve.

In the validation cohort, the AUC was 0.647 (95% CI: 0.518, 0.763), and the sensitivity, specificity, PPV, and NPV of the nomogram model were 81.82% (95% CI: 59.7%, 94.8%), 47.64% (95% CI: 32.0%, 63.6%), 45.0% (95% CI: 29.3%, 61.5%), and 83.3% (95% CI: 62.6%, 95.3%), respectively (Table 3).

### Prognostic prediction value of hepatic hilar LNs

In the validation cohort, a new regression equation (Equation B) was established by adding a novel variable, the size of the enlarged hepatic hilar LNs, into the nomogram model in the multivariate logistic regression analysis (Table 4). Equation B was formatted as follows: Log(*p*) = −2.395 + 1.573 × (LNs) + 1.419 × (Nomogram). In Equation B, the size of LNs was defined as 0 when the largest LN diameter was less than 0.8 cm and as 1 when it was more than 0.8 cm, and Nomogram was defined as 0 when the sum of nomogram total points was ≤100 and as 1 when it was >100. The ROC curves of Equation B and the nomogram for predicting KPE outcome in the validation cohort are shown in Figure 2C. The AUC, sensitivity, specificity, PPV, and NPV of Equation B were 0.798 (95% CI: 0.679, 0.888), 68.2% (95% CI: 45.1%, 86.1%), 83.3% (95% CI: 68.6%, 93.0%), 68.2% (95% CI: 45.1%, 86.1%), and 83.3% (95% CI: 68.6%, 93.0%), respectively. Of note, the AUC of Equation B was significantly greater than that of the nomogram (0.798 vs. 0.647, *P *= 0.042) in the validation cohort (Figure 2C).

**Table 4:** Multivariate regression analysis in the validation cohort.

	B	S.E	Wald	*P*-value	OR
Nomogram	1.419	0.670	4.486	0.034	4.134
LNs	1.573	0.593	7.043	0.008	4.820
Constant	−2.395	0.068	12.364	0.000	0.091

B, β coefficient; SE, standard error; Wald, *χ*^2^ value of Wald test; OR, odds ratio; LNs, lymph nodes.

## Discussion

In the present study, we established a nomogram consisting of three pre-KPE indicators (i.e., the gallbladder morphology on US, serum TBIL, and TP levels). The nomogram was validated to be useful in predicting the levels of TBIL at 3-month post-KPE in both the training and validation cohorts. Moreover, the present study showed that the predictive performance of the nomogram could be further improved with the addition of a novel indicator, the pre-KPE hepatic hilar LNs. Previous studies have demonstrated that the levels of TBIL 3-month post-KPE correlate well with the long term NLS of BA patients after KPE. Thus, the findings of the present study indicate that the outcomes of post-KPE might be accurately predicted by the nomogram based on the grey-scale US features and routine biomarkers, which are of significant clinical implications.

BA is a progressive, inflammatory, fibro-obliterative cholangiopathy that affects the hepatic ducts. The gallbladder is an important and the most easily observed part of the extrahepatic ducts. Thus, it is rational to hypothesize that the absence or morphological abnormality of the gallbladder might be a reflection of the fibro-obliterative severity of BA. In the present study, multivariate logistic regression analysis showed that the gallbladder was weighted highly in Equation A, implying its important role in the prediction equation, which confirms the hypothesis. Both TBIL and TP are routine serum markers used to test the liver function. The TBIL level is an important serum indicator that directly correlates with the severity of cholestasis, which also reflects the fibro-obliterative severity of the hepatic ducts. In addition, the serum TP level is an important indicator that can reflect physiological function of the liver and nutritional status of human body. The lower TP level implies more severe fibrosis and weaker physical conditions, which are likely to influence the outcome of KPE.

Previously, Zhang et al., evaluated a nomogram to predict the clearance of jaundice post-KPE (53). They demonstrated that the AUCs of their nomogram were 0.96 and 0.91, respectively, in the training and validation cohorts. In the present study, the AUCs of the established nomogram model in the training and validation cohorts were 0.673 and 0.647, respectively. Moreover, the nomogram model successfully predicted the satisfactory and unsatisfactory outcomes in 63.3% (31/49) and 71.4% (80/112), respectively, of the training cohort, and in 47.6% (20/42) and 81.8% (18/22), respectively, of the validation cohort. Although the performance of our nomogram model seems to be not as good as the one reported by Zhang et al., it is notable that their nomogram consisted of various factors including cytomegalovirus IgM-positive status, GGT, the thickness of the fibrous portal plate, liver stiffness, and multiple episodes of cholangitis. Some of these predictors are not easily available in clinical practice, whereas all the three predictors in our nomogram model are commonly used parameters in hospitals and medical centers equipped with grey-scale ultrasonography. In addition, our nomogram model provides a new perspective in exploring prediction models for the prognosis of BA post KPE.

Moreover, this present study also showed that the predictive performance of the nomogram was significantly improved with the aid of the hepatic hilar LNs in the validation cohort. The size of enlarged hilar LNs was first introduced as a new US feature for the diagnosis of BA by our group in 2019 (52), which showed that the hepatic hilar LNs with bile-stained macrophages were found in 84.4% of BA patients, and the size of LNs was proportional to the serum TBIL level and independent of age. One study (51) illustrated that the main factor contributing to hilar LNs size was variable expansion of the interfollicular cortical lymphoid tissue plus reactive sinusoidal histiocytosis with a mixture of hematopoietic precursors and bile-stained macrophages. The reactivity of the hilar LNs was correlated with the time of liver transplantation after KPE. This might explain why the hepatic hilar LNs could predict the outcome of KPE. However, we should acknowledge that Equation B was constructed with only a small number of cases, and thus, the potential value of using the size of enlarged hilar LNs needs to be verified with a large-scale study. Thus, we recommend that the nomogram model established and validated in the present study be applied in clinical practice.

The main limitation of the present study was that there might be selection bias due to the retrospective nature of the present study. For example, there were significant differences in some baseline clinical characteristics such as gallbladder morphology between the training cohort and validation cohorts. Nevertheless, the nomogram model achieved a good performance in both the training and validation cohorts, indicating that the selection bias, if any, was limited. Another limitation is that instead of NSL, TBIL level at 3-month after KPE was used to define the outcome of KPE. It is goes without saying that TBIL level at 3-month after KPE is not 100% correlated with long time outcomes of KPE. However, the TBIL level at 3-month after KPE is correlated very well with NLS, which has been confirmed by many studies (21–25). because of these limitations, the findings of the present study need to be further validated by research teams from other institutions.

## Conclusion

A nomogram based on the pre-KPE gallbladder morphology and serum levels of TBIL and TP to predict the outcome of BA 3 months post-KPE is established. Moreover, the addition of the size of the enlarged hilar LNs into the nomogram further improves its predictive value.

## Data Availability

The original contributions presented in the study are included in the article/Supplementary Material, further inquiries can be directed to the corresponding author/s.
